# Advanced Organ-on-a-Chip Devices to Investigate Liver Multi-Organ Communication: Focus on Gut, Microbiota and Brain

**DOI:** 10.3390/bioengineering6040091

**Published:** 2019-09-28

**Authors:** Lucia Boeri, Luca Izzo, Lorenzo Sardelli, Marta Tunesi, Diego Albani, Carmen Giordano

**Affiliations:** 1 Department of Chemistry, Materials and Chemical Engineering “Giulio Natta”, Politecnico di Milano, Piazza Leonardo da Vinci 32, 20133 Milan, Italy; lucia.boeri@polimi.it (L.B.); luca.izzo@polimi.it (L.I.); lorenzo.sardelli@polimi.it (L.S.); marta.tunesi@polimi.it (M.T.); 2 Department of Neuroscience, Istituto di Ricerche Farmacologiche Mario Negri IRCCS, via Mario Negri 2, 20156 Milan, Italy; diego.albani@marionegri.it

**Keywords:** liver-on-a-chip, microbiota, gut-liver-brain communication, multi-organ-on-a-chip platform

## Abstract

The liver is a key organ that can communicate with many other districts of the human body. In the last few decades, much interest has focused on the interaction between the liver and the gut microbiota, with their reciprocal influence on biosynthesis pathways and the integrity the intestinal epithelial barrier. Dysbiosis or liver disorders lead to0 epithelial barrier dysfunction, altering membrane permeability to toxins. Clinical and experimental evidence shows that the permeability hence the delivery of neurotoxins such as LPS, ammonia and salsolinol contribute to neurological disorders. These findings suggested multi-organ communication between the gut microbiota, the liver and the brain. With a view to in vitro modeling this liver-based multi-organ communication, we describe the latest advanced liver-on-a-chip devices and discuss the need for new organ-on-a-chip platforms for in vitro modeling the in vivo multi-organ connection pathways in physiological and pathological situations.

## 1. Gut Microbiota-Liver Communication

According to the latest estimate, 40 trillion microorganisms inhabit the human body, forming the complex microbial community [1]. The gut microbiota is the most important, and is a natural source of metabolites, hormones and toxins that regulate not only the gut physiology, but also extra-intestinal organs such as the liver and brain [2]. Its properties make the microbiota a key element in organ intercommunication, and microbial composition has a determinant role in triggering pathophysiological changes.

Despite the relative stability of the composition of the gut microbiota over one’s lifetime, genetic and environmental factors may lead to a dysbiotic condition and thus to a pathological imbalance in the microbial composition. Diet, antibiotics and lifestyle are examples of determinants of microbial balance [3,4,5]. For instance, in response to an increase in fructose intake, microbial composition changes, with reductions in the abundance of the beneficial populations of *Eubacterium* and *Streptococcus* [6].

In recent years, researchers have regarded the gut microbiota not just as a passive community, but as an active metabolic organ with precise, fundamental functions. Extensive work has led to the discovery that the microbiota plays key roles in a wide range of mechanisms, such as regulation of the immune response, maintenance of the intestinal barrier and energy homeostasis [7,8,9,10].

In terms of anatomy, the liver is the closest organ to the gastrointestinal tract and the gut microbial community with direct communication [11]. From the portal vein, the liver receives more than 70% of blood from the gut and the direct venous flow allows metabolite exchange between the gut and the liver. The molecules exchanged include pathogen-associated molecular patterns (PAMPs), such as lipopolysaccharides (LPS) produced by Gram-negative bacteria, and activate hepatic immune cells such as natural killer cells, Kupffer cells and hepatic stellate cells. In physiological conditions, this response has the regulatory role of controlling an excessive immune response to exogenous antigens. Metabolite exchange involves both microbiota products, such as short-chain fatty acids (SCFAs), and liver products such as primary bile acids (BA). The liver absorbs SCFAs mainly for lipid or glucogenesis. It regulates several mechanisms by activating specific proteins such as the carbohydrate-responsive element-binding protein (ChREBP) and G protein-coupled receptors [12,13]. The liver also produces BA, which the gut microbiota metabolizes to hydrophobic secondary BA that the portal vein reimports to the liver. Here BA regulates the absorption of fat and cholesterol, as well as several physiological processes such as lipid and glucose homeostasis. Dysbiosis alters BA metabolism and is associated with pathological conditions (e.g., non-alcoholic steatohepatitis) [14].

## 2. Integrity of the Intestinal Epithelial Barrier: The Influence of the Liver 

The intestinal physicochemical barrier (the mucosa) is a determinant in liver-based organ intercommunication, since it actively filters several molecules secreted by bacteria, like SCFAs and LPS, and prevents infections of the epithelium [15]. In the hepatic context, researchers have studied the intestinal barrier under different conditions. They have examined the direct effects of hepatic dysfunctions on the maintenance of barrier properties (e.g., permeability), and the changes induced by liver disorders on the composition of gut microbiota, focusing on how the secreted toxins affect the barrier integrity.

The mucosal surface is a complex structure where mucus and cells cooperate to protect the gut. Mucus is a biological hydrogel with an anisotropic architecture composed of an inner attached layer with no bacteria and loose outer layer where bacteria live. The anisotropy of the mucin network helps preventing infections and selectively regulates the passage of biomolecules from the lumen to the epithelium and vice versa. The thickness of the mucus layers varies along the gastrointestinal tract and depends closely on the state of health or disease. For instance, the mucus is 134 µm thick in the healthy colon, but can increase up to 232 µm during chronic inflammation [16]. Surprisingly, in patients with an alcohol-rich diet the mucus is 10 times thicker than in healthy subjects [17]. The mechanism is still not well understood, but it is believed to alter the entire process of mucus production, from synthesis to packaging. This leads to pathological down-regulation of anti-microbial molecules, such as regenerating islet-derived 3 beta (Reg3b) and regenerating islet-derived 3 gamma (Reg3g) proteins [17], and thus permits bacterial overgrowth and dysbiosis [18]. In an ex vivo mouse model of acute/chronic alcoholic feeding, the mucus hydrophilicity of the everted intestine decreased about 20% after ethanol exposure, with a significant reduction of the permeability of the mucus layer due to the loss of lipid content [19]. As lipids are the main components regulating the viscoelastic and diffusive properties of mucus [16], a deficiency in their content may lead to weakening of the mucus barrier and to “leaky gut”, which is a preliminary stage in alcoholic hepatitis and cirrhosis [20].

Alcohol also induces significant changes in the cellular components of the mucosa barrier, i.e., the epithelium. After alcoholic feeding, samples of human intestine presented morphological changes, such as myelin-like agglomerates in mitochondria and dilated endoplasmic reticulum [21]. In liver disorders transmembrane proteins forming the cellular tight junctions (occludins, claudins, cytoplasmic zona occludens proteins (ZO) and junctional adhesion molecules (JAM)), which have a key role in the barrier function of epithelium, are shattered, increasing cell permeability [22,23].

Alcoholic dependence is particularly aggressive in influencing the integrity of the tight junctions, because it directly damages cell-cell junctions and enhances the production of toxic molecules such as acetaldehyde and non-oxidative metabolites [22]. For instance, in a recent combined in vitro-in vivo study ethanol induced barrier dysfunction and liver injury with an epidermal growth factor receptor (EGFR)-dependent mechanism. Reduction of transepithelial electrical resistance (TEER), inulin-permeability and the disruption of the tight junctions indicated that both the integrity and permeability of the gut barrier were compromised [23]. Leclercq and colleagues reported that in patients psychological markers of alcoholic dependence (e.g., anxiety, depression and alcohol craving), as well as detoxification efficacy, correlate with the degree of gut permeability [24].

Pathological changes in microbiota composition were associated with inflammation, cytokine, chemokine and LPS production, tight-junction disruption and endotoxemia in an alcoholic mouse model [22]. These findings have suggested that the permeability of the gut barrier is negatively related to the total number of bacteria (in particular *Faecalibacterium prausnitzii*) and the dysbiotic condition of the mucosa in alcoholic patients, with a significant impact also on the efficacy of detoxification programs [24].

## 3. Gut-Liver-Brain Communication

Since the demonstration of the existence of the microbiota-liver axis, it has become interesting to study how it influences the rest of the human organism and the functions of other organs. Several studies have reported remarkable links between the liver, the gut microbiota and the brain.

Both chronic and acute alcohol rise stimulates the production of LPS [25], and alters the integrity of the gut epithelium and liver function. After crossing the gut barrier, LPS can be transported through the portal vein or lymphatic vessels to the liver. In physiological conditions, the liver is able to control the pathogenic stimuli and remove toxic compounds [26]. In pathological conditions however, this detoxification is partially lost and the unremoved LPS fraction enters the systemic circulation [27]. However, because of the lack of detoxification organs other than the liver, lymphatic transport remains the main LPS dissemination route. LPS release mainly affects on the inflammatory response in the whole organism; for instance causing neuroinflammation and brain damage [28]. At low concentrations, LPS cannot cross the blood-brain barrier (BBB), but it stimulates the production of pro-inflammatory cytokines, inhibits neurogenesis and reduces the brain volume. Otherwise, high levels of circulating LPS cause partial BBB disruption, so the toxin can then reach some cerebral regions, such as the cortex and thalamus [29].

LPS crosses the BBB in several neurodegenerative disorders, such as Alzheimer’s and Parkinson’s diseases [28,30]. The mechanisms underlying the actions and effects of LPS on brain tissue are still under investigation, but it is beyond a doubt that the brain, the microbiota, the gut and the liver have to be considered a single interconnected system.

Another mechanism that highlights the existence of gut-liver-brain communication is the production of the neurotoxin salsolinol. This tetrahydroisoquinoline derives from the reaction of dopamine with acetaldehyde (a derivative of ethanol) and is secreted by *Escherichia coli* in the presence of high levels of alcohol [31]. It is involved in the etiology of neurodegenerative disorders such as Parkinson’s disease. It can cross the BBB and increase oxidative stress and α-synuclein aggregation via cytochrome C oxidation [32,33,34]. In addition, chronic alcohol use stimulates the production of highly reactive molecules (e.g., reactive oxygen species), leading to oxidative stress [35].

Hepatic encephalopathy (HE) is another clear example of an interconnection among liver function, microbial mediation and neurological integrity [2]. Patients with HE have liver failure and a wide spectrum of symptoms, affecting either movement or personality. The pathogenesis is complex and not fully understood, but blood ammonia levels, oxidative stress and inflammation are key triggers [36]. Microbiota plays a role in the pathogenesis and development of HE by producing oxindole and ammonia, and excessive concentrations can lead to coma and brain edema. Consequently, dysbiosis and microbial composition are important regulating factors in HE. Bajaj and colleagues studied the microbial composition in cirrhotic patients with or without HE and the effect of microbiota-targeted treatments. They found an association between a high level of ammonia-producing bacterial species, such as Alcaligenaceae and Fusobacteriaceae, and poor cognitive functions [37].

Since the integrity of the gut barrier influences the production of inflammatory cytokines, the correlations among microbiota, inflammatory factors and neurological impairment are a hot topic for further investigations. Bajaj and colleagues have published other important results about possible microbiota-targeted treatments. They reported that fecal microbiota transplantated in HE patients improved cognitive functions and showed HE progression compared to control HE patients [38]. They have also indicated that withdrawal lactulose (a pre-biotic used to treat HE) withdrawal, acts on microbiota by modulating its functions, and not by altering its composition [39].

These recent clinical and experimental findings strongly support the idea of microbiota-liver-brain communication, with the liver acting as one of the main actors in the gut-brain axis [40,41].

To study in vitro the microbiota-liver-brain communication, each system needs to be accurately modeled and combined. This review focuses on describing the liver-on-a-chip devices developed to model and deepen our understanding of the hepatic physiology and function, or the more complex liver-based multi-organ communication. In the gut microbiota and the brain, research has made many advances in engineering in vitro 3D cell culture systems.

The gut has been widely modeled using advanced microfluidic organs-on-chips. In a very interesting review, Bein and colleagues described different microfluidic models, their complexity and potential [42]. They distinguished perfused membrane-based or membrane-free cell monolayer [43,44] from more complex mechanically active gut chips, able to mimic peristaltic movement [45]. Furthermore, to better study pathological conditions [46] such as inflammatory bowel disease (IBD), or to develop therapeutical strategies, they described microfluidic models implemented with microbiota culture systems.

A recent innovative engineered model was described by Jalili-Firoozinezhad and colleagues [47]. They developed a dynamic intestine-on-a-chip: (1) Hosting a model of the intestinal epithelium able to produce mucus; (2) culturing both anaerobic and aerobic microorganisms maintained at the same biodiversity level as in the human intestine; (3) equipped with oxygen sensors to monitor oxygen gradients, that are present in the human intestinal epithelium in physiological conditions.

Similarly, researchers have engineered neural cell culture systems to reproduce the complicated cerebral environment, comprising the BBB and the different cell populations—neurons and glial cells [48,49,50]. As for the liver and the gut, the strategies most considered so far are organ-on-a-chip devices. These can be adapted to the organ-specific requirements and provide rapid results comparable to in vivo conditions. For instance, in BBB models they implemented microfluidic organ-on-a-chip technologies using specific cell types—such as pericytes, astrocytes and endothelial cells—and measured BBB-related parameters such as barrier permeability and TEER [50,51].

## 4. Devices to Model the Liver: Features and Cell Components

The anatomical complexity and the key role of the liver in multi-organ communication means that liver in vitro modeling is still challenging. Current organ-on-a-chip devices offer the best solution to tackle anatomical complexity with suitable technological means [52]. Detailed analysis of the state-of-the-art reveals a booming development of innovative devices to model the liver. Table 1 reports the liver-on-a-chip devices to study single hepatic functions or liver multi-organ connections. It also summarizes the cell models used, which, as we will discuss below, are of key importance for a reliable model.

In the last few years, these miniaturized technologies have overcome the limitations of 2D in vitro cultures. In fact, the 2D hepatocyte monolayers generally used to reproduce hepatic functions are not exposed to the complex extracellular matrix and intercellular interactions of their native in vivo microenvironment. Furthermore, cell monolayers rapidly change their phenotypic properties and have limited survival [70]. Tissue slices represent a possible strategy to overcome the limitation of two-dimensionality maintaining the 3D architectural organization of physiological tissues. However, this in vitro model suffers poor availability and rapid phenotypic changes [70]. Organ-on-a-chip-based devices potentially make it possible to mimic the native hepatic environment better. They can host 3D cell constructs and perfuse with a continuous flow of fresh medium, while allowing real-time and non-invasive microscope monitoring [71].

No device efficiently reproducing all the liver’s functions and properties is available yet, but research is starting to open the doors to organ-on-a-chip platforms that will serve as an in vitro model of a complete liver.

**Figure 1:**
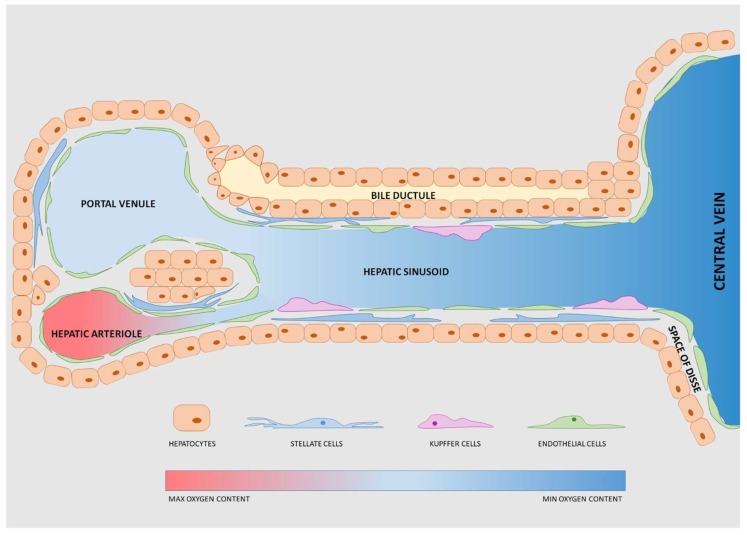
Schematic representation of the liver lobule. Each lobule has repeated functional units each with a portal venule and a hepatic arteriole, both directed into the central vein. The bile ductule transports the bile excreted by hepatocytes. Stellate cells, Kupffer cells and endothelial cells are defined as “non- parenchymal cells”.

The basic structure mostly addressed is the liver’s anatomical and functional unit: The lobule (Figure 1). This is a hexagonal unit where each edge has a portal triad (i.e., portal arteriole, portal venule and bile duct, all close together). The blood, from both the hepatic arteriole and the hepatic venule, mixes in a common channel called the hepatic sinusoid, which converges in the central hepatic lobule vein at the center of the lobule [72]. The hepatic sinusoid is surrounded by endothelial cells, forming a fenestrated wall inducing fast diffusion of nutrients, signaling factors or drugs [73,74]. Inside the sinusoid, the Kupffer cells (hepatic macrophages) break down damaged red blood cells [74], mediate antigen sensing and intercellular communication [75]. The hepatic cells form the liver parenchyma (about 60% of the liver volume) [52] and absorb/secrete molecules in the space of Disse, a peri-sinusoidal space separating hepatic cells from sinusoidal endothelial cells. This space also hosts the hepatic stellate cells involved in the metabolism of vitamin A and collagen synthesis [76]. Endothelial, Kupffer and stellate cells are defined as “non-parenchymal cells” (NPC) and constitute the remaining approximately 40% of the liver volume [52].

For accurate modeling of the complex hepatic structures and physiological processes, both cell source and culture environment are fundamental. To identify the best cell source, researchers have to compare its features with the specifications required for modeling of the system under investigation. The ideal cell source should: (1) Exhibit a complete hepatic function, such as the ability to metabolize endogenous and exogenous molecules; (2) maintain the differentiated state of hepatic cells and (3) be available on large scale [77].

Considering these parameters, the preferred cell source for liver-on-chips are primary cells from mammalian donors. They reproduce the complete hepatic function and offer results more comparable to in vivo conditions. However, these cells can be isolated only from whole or resected livers, so they are difficult to obtain. Furthermore, differences between donors generate inter-donor variability and poor experimental reproducibility.

Another limiting feature of primary cells is that during harvesting they start a de-differentiation process, due to the loss of cell-cell and cell-matrix interactions [78]. To overcome this drawback, some studies have tried altering the 3D environment and exploiting the crosstalk between NPC and hepatocytes [79].

Liver cell lines and stem cells are two other sources that offer interesting alternatives to primary cells, mainly due to their availability. To model hepatocytes, commonly used cell lines are HepG2, HepaRG or HepG2/C3A cells [80], while TMNK-1, HMEC-1 and LX-2 are the most used NPC-like lines [45,72]. The main disadvantage of using cell lines is their limited functional performance. For instance, the hepatoma cell line HepG2 is suitable for toxicological tests, but unsuitable to investigate specific hepatic metabolic pathways. On the other hand, respect to primary cells, the hepatoma HepaRG cells show higher differentiation markers, similar functions but lower responses to hepatotoxic drugs [81,82].

Stem cells can be distinguished as (1) adult stem cells, easy to harvest and able to differentiate into hepatocytes; and (2) pluripotent stem cells able to differentiate into multiple lineages [77]. While adult stem cells show some limitations related to epigenetic memory, and thus possible partial hepatic differentiation, pluripotent stem cells differentiate into all three germ layers, with unlimited growth. Researchers have used two types of pluripotent stem cells to model liver functions: Human embryonic stem cell (hESCs) and induced pluripotent stem cells (iPSC)-derived hepatocyte-like cells (HLC) [83]. Ethical issues about embryo manipulation limit the use of hESCs, but iPSC-derived HLC have no these ethical restrictions. Though they model immature hepatocytes (and thus are not representative of physiological hepatocytes), they require additional maturation methods to reach a level of maturation suitable for application in organ-on-a-chip devices [84].

To improve cell function and physiology, 3D cell culture models have been proposed, including spheroids and hydrogels embedding the co-culture of hepatic and NPC [52]. Bhise and colleagues investigated a combination of these two approaches by culturing hydrogel-embedded hepatocyte spheroids in a microfluidic system for 30 days [58]. They measured the oxygen concentration and the production of hepatic biochemical markers and their system was suitable for drug toxicity screening. Surprisingly, this organ-on-a-chip device gave results comparable to animal models, serving as a valid alternative to reduce, refine and replace in vivo measurements (3Rs principle).

**Figure 2:**
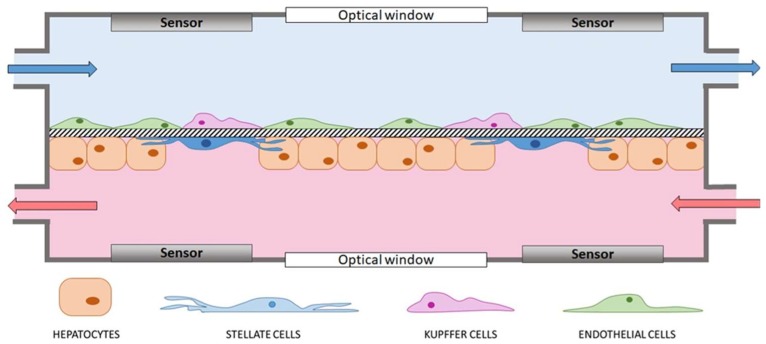
Schematic representation of an ideal lab-on-a-chip device modeling the liver sinusoid and based on hepatocytes, stellate, endothelial and Kupffer cells. A permeable membrane as thick as the space of Disse separates endothelial and Kupffer cells from hepatocytes and stellate cells. Culture medium continuously flows in the two compartments and the permeability of the membrane allows molecule diffusion from one chamber to the other. Sensors for real-time analyses (e.g., electrodes for transepithelial electrical resistance (TEER) measurements) equip both compartments, which have optical windows to ensure optical accessibility.

The most advanced liver-on-a-chip platforms should share the following features (Figure 2):

- Possibility to host 3D constructs and co-culture different cell types to reproduce the architectural organization of the hepatic microenvironment and model intercellular interactions. For instance, hydrogel-embedding cells or permeable membranes hosting the co-culture of hepatocytes and NPC are possible strategies to achieve these goals;
- Continuous perfusion of fresh medium to mimic the physiological blood flow and the high-rate exchange of molecules of the liver lobule. Moreover, well-determined shear stresses induced by a constant perfusion stimulate cell growth, proliferation and differentiation [46];
- Optical accessibility to allow real-time monitoring of the cells in culture;
- Sensors (e.g., electrodes and oxygen sensors) to measure the pivotal properties of the tissue (e.g., TEER or oxygen gradient, respectively) and evaluate the quality of the proposed model with respect to the native hepatic tissue. Sensors are also fundamental for comprehensive, non-invasive, real-time analysis of cell constructs during perfusion [52].

On-chip sensors have seen great developments in the last few years since non-invasive and live measurements have become a major need for complex 3D cell culture systems.

The main features required for the sensors are the:

- Ability to make non-localized measurements, to obtain values as representative as possible of the whole cell construct surface;
- Maintenance of optical accessibility;
- Biocompatibility of components materials and no release of toxic leachable in the medium;
- No noises or bias during the measure;
- Non-invasiveness for the cell constructs.

The sensors usually equipping liver-on-chips are oxygen, TEER and biochemical sensors. 

Oxygen sensors measure oxygen gradients and alarm the user if a too low oxygen concentration occurs in the culture chambers. To detect the oxygen consumption in each culture chamber, Rennert and co-workers applied oxygen sensors by a spray coating technique at both the inlet and outlet of the chamber. The sensors exploited the dynamic quenching principle of luminescence by molecular oxygen, but because of their positioning, no information about what was happening inside the chamber was available [53]. Domansky and colleagues proposed custom-made fiber optic probes. In their system, a 2-mm diameter ruthenium-based sensing layer (PreSens, Regensburg, Germany) detected oxygen levels thanks to the fact that the luminescence of ruthenium molecules in an excited state is quenched by collisions with molecular oxygen. The probes were connected to a four-channel fiber optic meter and luminescence decay time was measured by phase modulation. The sensing layers allowed for a direct measurement, because they were placed inside the chamber, in contact with culture medium. However, their read-outs were local and no information about oxygen distribution throughout the cell construct were available [65]. To efficiently fill this gap, Bavli and co-workers proposed tissue-embedded microparticles loaded with a ruthenium-based dye [54]. To avoid the invasiveness of tissue-embedded sensors, Bale and colleagues preferred a modified microfluidic advanced microphysiological system (MPS) incorporating strip-based oxygen sensing foils [60].

TEER electrodes measure the resistance that cells oppose to the passage of current through the cell construct. Two similar electrodes on both sides of the construct undergo a voltage potential difference that corresponds, coherently with Ohm’s law, to electrical resistance. The TEER comes out by multiplying this resistance by the surface area of the construct. To ensure conductivity and a stable measurement, AgCl or Au-Ag-Cr electrodes are preferred. The voltage difference is usually measured with commercially available instruments dedicated to the TEER, which is therefore an efficient indicator of the cell construct integrity: The lower the amount of cell-cell junctions, the lower the electrical resistance [44].

The biochemical sensors may be configured differently, depending on the compound to be measured. For instance, to assess the paracrine crosstalk between hepatocytes and stellate cell lines after alcohol injury, Stenken and Poschenrieder equipped microfluidic co-cultures with biosensors for continuous monitoring of transforming growth factor beta (TGF-β) [85]. Similarly, to assess drug-induced liver injury in vitro, Prill and co-workers used electrochemical sensors exploiting the enzymatic reactions of glucose oxidase (GOD) and lactate oxidase (LOD) [86].

Since the liver has multiple roles and is involved in the physiology and pathology of different organs (besides the gut and the brain), the development of multi-organ models networking the liver and other specific organs is of considerable interest [87].

### 4.1. Single Liver-on-a-Chip Devices

To study the shift from mitochondrial respiration to glycolysis after mitochondrial damage, Bavli and colleagues developed a microfluidic system able to culture growth-arrested HepG2/C3A cell-based spheroids under physiological conditions for 28 days [54]. From the bottom to the top, it had a poly-(methyl methacrylate) (PMMA) housing, a glass cover, nine laser-cut polydimethylsiloxane (PDMS) microwells for spheroid culture, a glass window at the top for optical access and a PMMA cover with locking screws for a hydraulic seal. It also allowed for real-time monitoring of oxygen, glucose and lactate concentrations. The maximum computed shear stress was 0.03 Pa at a flow rate of 2 μL/min [54]. However, a disadvantage of this system is the inclusion of only one cell line modeling hepatocytes, limiting its reliability to simple applications and live observations.

Rennert and co-workers developed the first microfluidic device (microfluidic organ tissue flow, MOTiF) showing morphological and functional similarities with the human liver and including NPC and oxygen sensors [53]. Their device resembled the liver sinusoid and mimicked both the cell composition and organization. It allowed cellular interactions and the exchange of paracrine signals from cell to cell. It had a vascular and a hepatic cell layer, obtained with a double seeding procedure. The vascular layer was composed of human umbilical vein endothelial cells (HUVEC) and primary macrophages, while the co-culture of HepaRG hepatocytes and LX-2 stellate hepatic cells formed the hepatic layer. A polyethylene terephthalate (PET) microporous membrane (8 μm pore diameter) in the middle of the two cell layers acted as a cell substrate and modeled the fenestration of the space of Disse. The perfusion using a syringe pump offered two different flow rates in the hepatic and vascular layers. Since hepatic cells tolerate only very low shear stresses [53], the authors opted for a very low flow rate in the hepatic layer and a higher flow rate in the vascular layer. For this reason, they reached the physiological flow rate (50 µL/min) only on the vascular layer. To reproduce the physiological flow of the hepatic layer, a possible strategy could be the encapsulation of the hepatic cells inside spheroids or polymer-based materials, to protect them from high shear stress values.

To this goal, in 2018 Ma and colleagues developed a 3D microfluidic spheroid-based liver model (3D-LOC) to mimic the structure of the human hepatic sinusoid [62]. The device had a PMMA base including a microscope coverglass and a microwell layer with 1800 holes for the culture of hepatic spheroids. To model the fenestrated sinusoid wall, the authors added a Transwell^®^ insert with a microporous membrane over the microwell layer and designed a microfluidic channel (2000 μm wide and 200 μm high) over the membrane for medium flow. Three thumbscrews locked a PMMA lid to two steel tubes (inlet and outlet channels) to ensure hydraulic sealing. In the assembled device, the membrane divided the upper zone, defined by a microfluidic chamber with high shear stresses due to medium flow, from a lower zone for spheroid culture. On the microwell side, the glass support allowed optical access. A peristaltic pump with debubblers connected in series automated the perfusion and ensured the simultaneous working of four identical units. However, the absence of NPC and sensors is a major drawback of this system [62].

Another interesting work, published by Yu and co-workers, described a perfusion-incubator-liver-chip (PIC) for 3D cell culture with applications for hepatotoxicity testing [56]. In this system, hepatocyte spheroids experience optimal mass transfer and limited shear stress and maintained their viability for over 24 days. It combined biocompatible materials (a rigid, well-defined, reusable glass/silicon structure with elastic and gas-permeable PDMS assemblies) with a low capacity for small molecule absorption. The chip structure consisted of three main elements: A glass/silicon structure including the microfluidic chamber, a cell culture support for insertion of the spheroids and a PDMS/glass seal closing the microfluidic circuit and removing bubbles from the circuit (thus reducing the shear stress on hepatocytes). To eliminate the need for a cell incubator, but not the precision over control of the medium temperature in the culture chamber, the authors added a heater, together with a temperature controller and a thermocouple.

As a whole, the solutions presented could open the doors to the development of hepatic cell models for long-term experiments. However, more technological tools and complex platforms are required for the culture and monitoring of human primary hepatocytes and NPC.

### 4.2. Liver-Based Multi-Organ-on-a-Chip Platforms

The liver is dedicated to the metabolism of molecules and drugs targeting other human districts, with a huge impact on the physiology and functions of other organs. To understand its multi-organ cross-talks, the liver has been modeled and included in multi-organ-on-a-chip platforms. These innovative systems interconnect two or more organs to simulate the systemic organ recirculation. For instance, in 2016 Esch and colleagues developed a modular, pumpless body-on-a-chip platform and co-cultured gastrointestinal (GI) and liver tissue for 14 days [67]. Since the two tissues require different conditions, initially they were matured on a single organ-on-a-chip, and then combined in a single device. From the top to the bottom, the platform had five layers: The top lid contained the fluidic channel for the GI tract, then there was a porous membrane seeded with the epithelial cell line Caco-2, a lid containing the fluidic channel for the liver, a 3D scaffold for hepatocytes and NPC, and finally the base at the bottom. The fluidic channels for the GI tract perfused the apical side of the GI epithelium, while the liver chip contained a fluidic channel that perfused the liver chamber as well as the basolateral side of the GI epithelium. A porous membrane allowing for the exchange of soluble metabolites connected the GI epithelium and the liver. The force of gravity (together with a sophisticated passive valve mechanism) moved the medium across both chambers. Two Ag/AgCl electrodes equipped the superior and inferior chambers to measure the TEER [67]. This complex system maintained cells in culture for 14 days, but lacked an automatic, continuous flow of fresh medium and optical accessibility. Despite these limitations, it paved the way to future versatile platforms where several compartments can be connected to model human tissues in vitro.

In 2018 Theobald and co-workers developed a multi-organ-on-a-chip device modeling both liver and kidney tissue to mimic the metabolism of vitamin D3 (25(OH)D3). The device had a simple structure, with two chambers for 2D cell culture (HepG2 cells for the liver model and primary renal proximal tubule epithelial cells for the kidney model) in series. Vitamin D3 was dissolved in the medium flowing in the microfluidic system. Initially, it was assimilated by the liver cells, then transported to the kidney chamber, where it was metabolized to 1,25(OH)2D3. After treatment with the eluted 1,25(OH)2D3, HL-60 human leukemia cells showed a pro-differentiation effect confirmed by the expression of differentiation markers [69]. Theobald and colleagues proposed an optically accessible device and subjected the cells to a continuous flow of fresh medium. Its modularity could be adapted to different cell models, but they must share the same culture medium. Differently from the device proposed by Esch and colleagues, the model by Theobald and colleagues did not have electrodes and TEER measurements were not possible.

In the same year, Bovard and co-workers developed a lung-liver-on-a-chip for acute and chronic toxicity studies on aerosols [68]. Their platform could be placed in an incubator and had four main parts: The microfluidic chip for cell culture, a reservoir plate for medium collection, a pumping unit for medium perfusion and a smartphone for remote control. The chip and the reservoir plate were made up of poly-etheretherketone (PEEK), a non-absorbent medical material allowing the long-term culture (about 28 days) of spheroid-based cell models. The fluidic plate had four identical microfluidic circuits able to work simultaneously. Each circuit contained two identical cell culture wells connected by a small channel (2.5 mm diameter). The first well contained normal human bronchial epithelial tissue at the air-liquid interface, while the second was available for HepaRG cell spheroids. The compartment hosting the lung model had a small cavity on the bottom for a Transwell^®^ insert. In the liver compartment, the bottom contained three concentric grooves 1 mm deep to segregate the spheroids. The system was optically accessible and compatible with manual measurements of the TEER with chopstick electrodes [60]. The pumping system ensured automatic recirculation of the medium while a manual medium refresh was necessary every day. Despite the multi-organ approach, this device (as well as the model by Theobald and colleagues) did not exploit NPC and failed to fully represent liver physiology.

## 5. Present and Future Prospects

As described, the most recent platforms have several aspects still lacking to model the liver itself and liver-based multi-organ communication. Above all, some organ intercommunications—such as the gut-liver-brain interconnection—are fully unexplored. The advantages of the devices developed so far and a multi-organ platform modeling the liver as an active element communicating with the epithelial barrier and indirectly with the brain, could be combined to study in vitro the liver-mediated communication between the gut and the brain. For instance, the device proposed by Esch and colleagues could be made optically accessible and continuously perfused with fresh medium, as in Theobald’s system [58,60]. To examine the effects of the liver on the integrity of the epithelial barrier, the system should be implemented with the possibility of measuring the TEER, as in Esch’s and Bovard’s systems [59,60]. Finally, to integrate the interconnection with the brain, the device could be expanded, by adding, for instance, a third microfluidic chamber in series for neuronal cells.

An intriguing complex multi-organ-on-a-chip-based platform has been designed to model the microbiota-gut-brain axis within the MINERVA project (www.minerva.polimi.it), funded by the European Research Council (ERC). The main aim of this multi-organ device is to investigate the effects of the molecules released by the gut microbiota on brain pathophysiology. The platform is based on five miniaturized and optically accessible microfluidic organ-on-a-chip devices connected sequentially and designed to represent the main key players of the microbiota-gut-brain axis: The microbiota, the gut epithelial barrier, the immune system, the blood-brain-barrier and the brain. Each device hosts three chambers with advanced cell-based models and integrated electrodes to measure the electrical properties of biological barriers, such as the gut epithelium and the blood-brain barrier.

The MINERVA platform stems from a miniaturized optically accessible bioreactor developed in 2012 [88], which has been successfully used for several applications in fields ranging from neuroscience to cancer [89,90,91]. With its versatile design, MINERVA will be implemented to host another organ-on-a-chip specific for the liver, connected with the gut epithelial barrier compartment. This should allow the study of communications between the gut microbiota and the liver, for instance, to assess the influence of the liver on the epithelial barrier’s integrity and its permeability to neurotoxins, such as oxindole and salsolinol, potentially able to trigger brain pathology.

## Figures and Tables

**Table 1:** Advanced liver-on-a-chip devices to study hepatic functions and liver-based multi-organ communication [53,54,55,56,57,58,59,60,61,62,63,64,65,66,67,68,69].

Device	Cell Models	Ref.
*Single liver-on-a-chip devices*		
Microfluidic organ tissue flow (MOTiF) resembling a three-dimensional human liver model.	LX-2 cell line + HepaRG cell line + HUVEC cell line + primary macrophages	[53]
Microfluidic spheroids culture system under physiological conditions.	HepG2/C3A cell line	[54]
Liver-on-a-chip with bioprinted constructs for drug screening applications.	HepG2/C3A cell line organized in spheroids suspended in hydrogel	[55]
Perfusion-incubator-liver-chip (PIC) for spheroids culture with application in hepatotoxicity testing	3D rat primary hepatocyte spheroids	[56]
Liver-on-a-chip to study hepatitis B virus infection	Primary human hepatocytes both monocultured and co-cultrured with Kuppfer cells.	[57]
Microfluidic system for hydrogel-embedded cell spheroids culture	HepG2/C3A cell line	[58]
PRDEICT-96 array: a thermoplastic oxygen-permeable microfluidic system designed as a 96-well microfluidic array with a recirculating pumping system	Primary human hepatocytes	[59]
Microfluidic bilayer model with thermoplastic materials used to study liver diseases, cellular interactions and therapeutic responses.	Primary human hepatocytes both monocultured and cocultured with Kuppfer cells.	[60]
Microfluidic device to investigate non-alcoholic fatty liver disease (NAFLD).	HepG2/C3A cell line	[61]
Three-dimensional microfluidic spheroid-based liver model (3D-LOC) to mimic the human hepatic sinusoid structure.	HepG2/C3A cell line	[62]
Microengineered bioartificial liver for drug toxicity screening.	Mouse hepatocyte line (H-4-II-E) and primary mouse hepatocytes.	[63]
Automated droplet device-based microfluidic platform for multiplexed analysis of biochemical markers in small volumes.	Primary rat hepatocytes organized in spheroids.	[64]
Multiple bioreactors integrated in an array that forces maintenance of 3D liver model culture under constant perfusion.	Primary rat hepatocytes and liver sinusoidal endothelial cells (LSEC) enriched with primary stellate and Kuppfer cells.	[65]
*Liver-based multi-organ-on-a-chip platforms*		
Liver-kidney co-culture biochip to investigate ifosfamide nephrotoxicity.	HepaRG/HepG2C3A cell line and MDCK cell line	[66]
Body-on-a-chip for the co-culture of gastrointestinal (GI) tract epithelium and three-dimensional primary liver.	Epithelial cell line Caco-2 + primary hepatocytes + primary NPCs	[67]
Multi organ-on-a-chip mimicking the interaction between lung and liver.	HepaRG cell line + primary normal human bronchial epithelial (NHBE) cells	[68]
Multi organ-on-a-chip mimicking the interaction between lung and kidney	HepG2 cell line + primary renal proximal tubule epithelial (RPTEC) cells	[69]
